# Double-Negative T Cells Regulate Hepatic Stellate Cell Activation to Promote Liver Fibrosis Progression *via* NLRP3

**DOI:** 10.3389/fimmu.2022.857116

**Published:** 2022-03-15

**Authors:** Yi Yang, Yongjia Sheng, Jin Wang, Xiaohong Zhou, Wenyan Li, Caiqun Zhang, Li Guo, Chenyang Han

**Affiliations:** ^1^ Department of Pharmacy, The Second Affiliated Hospital of Jiaxing University, Jiaxing, China; ^2^ Department of Neurology, The Second Affiliated Hospital of Jiaxing University, Jiaxing, China; ^3^ Department of Center Laboratory, The Second Affiliated Hospital of Jiaxing University, Jiaxing, China

**Keywords:** double-negative T cells, liver fibrosis, NLRP3, TNFR1, hepatic stellate cells

## Abstract

**Aim:**

We mainly explored the role and mechanism of double-negative T cells (DNTs) in liver fibrosis.

**Methods:**

DNTs were co-cultured with mouse hepatic stellate cells (HSCs). Later, cell viability was detected by Cell Counting Kit-8 (CCK-8) assay; α-SMA expression was measured through fluorescence staining; TNF-α, IL-6, and MMP-9 levels were measured by ELISA; and the expression of Bcl-2, TGF-β1, NLRP3, ASC, and TNFR1 proteins in HSCs was detected by Western blotting (WB) assay. At the same time, HSC-*NLRP3^−/−^* and HSC-*TNFR1^−/−^* are used to explore the mechanism. In mouse experiments, mice were intraperitoneally injected with DNTs; afterward, the hepatic tissue fibrosis degree was detected by Masson staining, α-SMA expression was measured through immunohistochemistry (IHC) assay, and histopathological changes were detected by sirius-red staining and H&E staining.

**Results:**

The results suggested that DNTs promoted HSC activation and NLRP3 activation. The effect of DNTs on activating HSC-*NLRP3^−/−^* was suppressed, and the difference was significant as compared with HSCs. HSC-*TNFR1^−/−^* activation was also inhibited. To explore the mechanism of DNT-secreted TNF-α in TNFR1-NLRP3 activation, we transfected DNTs with TNF-α siRNA; as a result, DNTs with TNF-α silencing did not significantly affect HSC activation. DNTs promoted hepatic tissue fibrosis progression and HSC activation; after treatment with NLRP3 inhibitor, the effect of DNTs on promoting fibrosis was suppressed.

**Conclusion:**

We discovered that DNTs played an important role in liver fibrosis and that DNTs promoted HSC activation *via* the TNF-α–TNFR1-NLRP3 signal axis, thus further promoting liver fibrosis progression.

## Background

In normal mouse peripheral lymphoid organs, a majority of αβ-TCR^+^ lymphocytes express CD4 or CD8 molecule, while just 1%–5% of them express αβ-TCR but not CD4 or CD8, and this portion of cells is called double-negative T cells (DNTs) (1, 2). Although extensive studies have been conducted on CD4^+^ and CD8^+^ T cells, the source of peripheral DNTs remains unclear so far. There may be multiple pathways involved in the formation of DNTs (3). As discovered in existing studies, DNTs can promote the progression of ischemic stroke through inflammatory response, and their effect is related to the secretion of multiple inflammatory factors (4, 5). Nonetheless, the role of DNTs in other diseases has not been reported.

Liver fibrosis is a kind of common liver disease, which is mainly caused by the hyperplasia and deposition of extracellular matrix (ECM) resulting from hepatic stellate cell (HSC) activation, as well as an imbalance between ECM synthesis and degradation (6, 7). HSC activation and hyperplasia are the central links in the occurrence and development of liver fibrosis (8), and suppressing HSC activation is the key to the prevention and treatment of liver fibrosis. The therapeutic measures of liver fibrosis mainly include the suppression of HSC proliferation or induction of HSC apoptosis (9), suppression of collagen production or promotion of collagen degradation, cytokine regulation, and mesenchymal stem cell (MSC) perfusion (10, 11). In HSC research, NLRP3 is an important factor that promotes HSC proliferation and activation (12, 13), but its activation mechanism remains unclear.

We discovered when detecting the peripheral blood of liver fibrosis patients that the proportion of DNTs (CD3^+^CD4^−^CD8^−^) in peripheral blood of liver fibrosis patients significantly increased, while that of CD3^+^TNF-α^+^ also increased. Based on the previously reported regulatory role of DNTs in an inflammatory response, this study aimed to reveal the mechanism of action of NLRP3 in liver fibrosis.

## Materials and Methods

### Detection of Double-Negative T Cell Proportion in Peripheral Blood Mononuclear Cells of Liver Fibrosis Patients

This study enrolled 25 liver fibrosis patients diagnosed at The Second Affiliated Hospital of Jiaxing University and 25 healthy subjects from the Physical Examination Center from January 2019 to January 2020. After informed consent was obtained from each patient, 8 ml of peripheral blood (elbow vein blood) was collected to extract peripheral blood mononuclear cells (PBMCs). Later, the samples were divided into two portions, one for the detection of DNT proportion and the other one for the detection of CD3^+^TNF-α^+^ proportion. Later, cells were incubated with monoclonal antibodies against PC5-CD3, FITC-CD4, and PE-CD8 (20 μl each; BD, Massachusetts, USA) at room temperature in dark for 15 min. After being washed with the pre-chilled phosphate-buffered saline (PBS), cells were centrifuged at 1,500 rpm, mixed with 0.5 ml of PBS, and detected using the EPICS XLII flow cytometer (Beckman-Coulter, California, USA), and the CD3^+^CD4^−^CD8^−^ cell proportion was analyzed with CellQuest software. Moreover, when detecting DNTs with monoclonal antibodies against PC5-CD3, FITC-CD4, PE-CD8, and APC-TNF-α (20 μl each), the proportion of CD3^+^TNF-α^+^ in DNTs was analyzed by CellQuest software.

### Role and Mechanism of Double-Negative T Cells in Inducing Hepatic Stellate Cell Activation

Mouse splenic DNTs were isolated and amplified by Rosettesep antibody adsorption and Easysep magnetic-activated cell sorting (MACS). Later, C57BL/6J mice were given an intraperitoneal injection of 0.25 mg/g of carbon tetrachloride to induce liver fibrosis once every 3 days for 6 consecutive weeks, and the mouse model of liver fibrosis was successfully constructed. Thereafter, mice were sacrificed through carbon dioxide suffocation; then the spleen was dissected and ground in liquid nitrogen to prepare the cell suspension. Afterward, the total lymphocytes were isolated using the lymphocyte separating medium (Dakewe Biotechnology Co., Ltd., Beijing, China). Later, cells were inoculated into the 24-well plates coated with anti-CD3mAb (100 ng/ml) and incubated overnight. After being washed thrice with PBS containing 2% fetal bovine serum (FBS), cells were cultured with Roswell Park Memorial Institute (RPMI)-1640 medium that contained 20% FBS (Sigma, Massachusetts, USA), and DNTs were obtained by MACS. The mouse HSCs were divided into the control group and DNT co-culture group. Cells in the DNT co-culture group were co-cultured in the Transwell chambers at the DNTs : HSCs = 1:3. The following assays were conducted.

#### Cell Viability Was Detected by Cell Counting Kit-8 Assay

At 48 h after culture, the medium of DNTs was isolated, and HSCs were inoculated into the 96-well plates. Both control and DNT groups were set, cells in the DNT group were cultured with the DNT medium, and those in the control group were cultured with a routine medium. Cell viability was detected at 12, 24, and 48 h after cell culture. Then, the medium was discarded, 100 μl of fresh medium was added into each well, cells were further incubated with 10 μl of Cell Counting Kit-8 (CCK-8) solution for 4 h, and absorbance [optical density (OD)] value was measured at 450 nm.

#### α-SMA Expression Was Detected by Immunofluorescence Staining

Cell slides were used for staining. To be specific, the sterile slides were added into the lower Transwell chamber and co-cultured with DNTs for 48 h after HSC inoculation. Later, HSCs were harvested, fixed with 4% formaldehyde at room temperature for 0.5 h, and permeabilized with 0.2% Triton X-100 for 5 min. After being washed with PBS thrice, cells were incubated with the α-SMA monoclonal antibody at 4°C overnight, washed with PBS twice, further incubated with fluorescence secondary antibody, mounted with 95% glycerin, and observed under the fluorescence microscope.

#### The Levels of TNF-α, IL-6, and MMP-9 Were Detected by ELISA

After DNTs were co-cultured with HSCs for 48 h, the medium and cells were collected separately. Then, cells were lysed with 100 μl of NP-40 lysate to extract the intracellular proteins; meanwhile, the medium was centrifuged to obtain the supernatant medium. In line with the ELISA kit (Nanjing Institute of Biological Engineering, Nanjing, China) instructions, the expression of TNF-α, IL-6, and MMP-9 in the medium was measured.

#### The Expression Levels of COL-I and COL-III Were Detected by ELISA

Intracellular proteins and proteins in the medium were extracted according to the abovementioned method; then the expression of COL-I and COL-III was detected by the ELISA kit (Abcam, Massachusetts, USA).

#### The Expression Levels of Bcl-2, TGF-β1, NLRP3, ASC, and TNFR1 Proteins Were Detected by Western Blotting Assay

The abovementioned cell protein liquid extracted after culture was used for Western blotting (WB) assay. In brief, the 8%–12% sodium dodecyl sulfate–polyacrylamide gel electrophoresis (SDS-PAGE) gel was prepared; then the protein liquid was diluted with 5× loading buffer to a volume of 20 μl. After being boiled for 8 min, the sample was loaded for electrophoresis at the voltage of 80 and then 120 V and transferred onto the membrane at the constant current of 300 mA for 0.5–2 h. Later, the membrane was blocked with 5% skimmed milk powder for 2 h and incubated with TBST-diluted monoclonal primary antibodies (1:400; Abcam, Massachusetts, USA) against Bcl-2, TGF-β1, NLRP3, ASC, and TNFR1. Afterward, the membrane was further incubated with horseradish peroxidase (HRP)-labeled goat anti-rabbit secondary antibody (Abcam, USA). Finally, the membrane was detected with chemiluminescence, and the OD value was analyzed using the Image Pro-Plus 6.0 software.

### Effect of Double-Negative T Cells on Hepatic Stellate Cell Activation After NLRP3 (HSC-*NLRP3^−/−^*) Knockdown

To investigate whether DNTs exerted their effect *via* NLRP3, we co-cultured HSCs with NLRP3 knockdown (HSC-*NLRP3^−/−^*) with DNTs. The effect on HSC activation was analyzed based on the abovementioned method.

### Effect of Double-Negative T Cells on Hepatic Stellate Cell Activation After TNFR1 (HSC-*TNFR1^−/−^*) Knockdown

DNTs are cells excreting a large amount of TNF-α. To investigate the reactivity of the TNFR1 signal in NLRP3 activation, we knocked down TNFR1 in HSCs to construct the TNFR1 knockdown HSCs (HSC-*TNFR1^−/−^*) and co-cultured them with DNTs to detect the effect on HSC activation.

### Effect on Hepatic Stellate Cell Activation After TNF-α Silencing in Double-Negative T Cells (TNF-α siRNA)

To investigate that DNTs secreted TNF-α to mediate TNFR1-NLRP3 and promote HSC activation, we silenced TNF-α expression with siRNA in DNTs. The target sequence of siRNA was GCG TGG AGC TGA GAG ATA A, the sense strand (5–3) was 5′-GCGUGG AGC UGA GAG AUA -3', and the antisense strand (3–5) was 3′ - CGC ACC UCG ACU CUC UAU U-5′. After siRNA transfection, DNTs were co-cultured with HSCs to detect the effect on HSC activation.

### Effect of Double-Negative T Cells on Liver Fibrosis Mice and Its Mechanism

The C57BL/6J mice were randomly divided into control, FIB (liver fibrosis), FIB^+^DNTs, and FIB^+^DNTs^+^MCC950 groups, with 10 in each group. In the mouse model of liver fibrosis, 0.25 mg/g of carbon tetrachloride was intraperitoneally injected into each mouse once every 3 days to induce liver fibrosis (14, 15). After injection for 6 weeks, the mouse model of liver fibrosis was successfully constructed. In the DNT group, mice were intraperitoneally injected with DNTs once every week and given intragastric administration of 10 mg/kg of MCC950 (the inhibitor of NLRP3) once for 6 weeks. After 6 weeks, mice were sacrificed through carbon dioxide suffocation, and peripheral blood and hepatic tissues were collected for detection.

#### Live Histopathology Was Detected by H&E Staining

Hepatic tissues were dehydrated with alcohol, transparentized, embedded in paraffin, and sliced into 4-μm sections. Later, sections were dried for preservation. After being dried, sections were deparaffinized with xylene twice, hydrated with alcohol, and stained with hematoxylin for 3 min. Afterward, sections were treated with 1% hydrochloric acid alcohol, stained with eosin for 5 min, dehydrated, transparentized with xylene, and mounted with neutral resin, followed by observation of liver pathological changes under the microscope.

#### Fibrosis Lesion of Hepatic Tissue Was Detected by Masson Staining

The tissue sections were treated according to the description in H&E staining. First of all, sections were stained with Masson for 5 min, with phosphomolybdic acid for 5 min, and with aniline blue for another 5 min. The sections were subsequently treated within the differentiation solution, hydrated with alcohol, transparentized with xylene, mounted with neural resin, and observed under a microscope for liver fibrosis level.

#### Collagen Fiber Level Within Hepatic Tissues Was Detected by Sirius-Red Staining

In brief, sections were deparaffinized, immersed in distilled water for 2 min, stained with Harris hematoxylin solution for 3–5 min, and washed with distilled water thrice. Later, sections were stained with sirius-red staining solution for 15–30 min, color-separated and dehydrated with absolute ethyl alcohol, transparentized with xylene, dried in the air, and mounted with neutral resin. Finally, the expression level of collagen fibers in hepatic tissues was observed under the microscope.

#### α-SMA Expression Was Detected by Immunohistochemistry Staining

The hepatic tissue sections were deparaffinized with xylene, immersed in absolute ethyl alcohol, and heated in an oven at 98°C for antigen repair. Later, the sections were incubated with 3% hydrogen peroxide for 10 min under ambient temperature to eliminate the endogenous peroxidase. Afterward, the sections were blocked with 2% bovine serum albumin (BSA) for 30 min at 37°C. Later, antigens were added to non-specifically bind to the antibody, and the primary antibody was the anti-α-SMA antibody (1:250, Abcam, USA). Subsequently, sections were further incubated with peroxidase-labeled streptomycin (Abcam, USA) for 15 min; then, the freshly prepared DAB solution (DAKO, Glostrup, Denmark) was added dropwise onto each section for color development. Afterward, sections were sufficiently washed with tap water, counterstained with hematoxylin, and sealed. All sections were finally observed under the Olympus-BX51 upright microscope equipped with Olympus-DP72 image collection system and CRi Nuance multispectral imaging system (Cambridge Research & Instrumentation, Hopkinton, MA, USA).

#### The Expression Levels of TNF-α, IL-6, MMP-9, COL-I, and COL-III in Peripheral Blood and Hepatic Tissues Were Detected by ELISA

In brief, mouse peripheral blood was centrifuged at 3,000 r/min for 30 min, and the supernatant was collected for subsequent use. The mouse hepatic tissues were ground with liquid nitrogen, and later tissue cells were lysed with NP-40 lysate to collect the supernatant. In line with ELISA kit instructions, the expression levels of TNF-α, IL-6, MMP-9, COL-I, and COL-III in peripheral blood and hepatic tissues were detected, and the results were expressed as pg/ml.

#### Protein Expression Levels Were Detected by Western Blotting Assay

The total protein was extracted from hepatic tissues according to the aforementioned method to detect the relative protein expression.

### Statistical Analysis

Statistical analysis was completed using SPSS19.0 software. Measurement data were expressed as mean ± SD $\left(\bar{\text{x}}\pm \text{s}\right)$ , one-way ANOVA was adopted for comparison among multiple groups, and Student–Newman–Keuls (SNK) test was conducted for comparison between groups. p < 0.05 indicated that the difference was of statistical significance.

## Results

### Proportion of Double-Negative T Cells in Peripheral Blood Mononuclear Cells of Liver Fibrosis Patients

**Figure 1:**
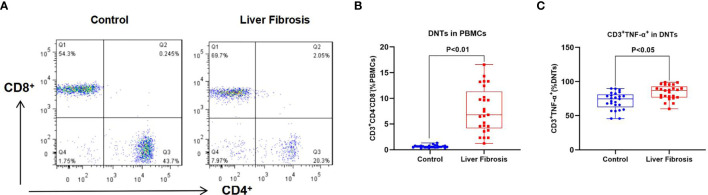
Proportion of DNTs in peripheral blood of liver fibrosis patients. **(A, B)** Proportion of DNTs in PBMCs. The proportion of DNTs in peripheral blood of liver fibrosis patients was remarkably higher than that in healthy controls. **(C)** Proportion of CD3^+^TNF-α^+^ cells in DNTs. The proportion of CD3^+^TNF-α^+^ cells in DNTs was evidently elevated in liver fibrosis patients, which was higher than that in healthy control, suggesting that TNF-α was highly expressed in DNTs of liver fibrosis patients. DNTs, double-negative T cells; PBMCs, peripheral blood mononuclear cells.

We discovered when detecting the proportion of DNTs in PBMCs of liver fibrosis patients that the proportion of DNTs in peripheral blood of liver fibrosis patients was significantly higher than that in healthy controls; meanwhile, the proportion of TNF-α-positive DNTs also significantly increased in liver fibrosis patients compared with healthy controls. This suggested that TNF-α was one of the important secretory factors of DNTs, which played a certain role (**Figure 1**).

### Effect of Double-Negative T Cells on Hepatic Stellate Cell Activation and Its Mechanism

**Figure 2:**
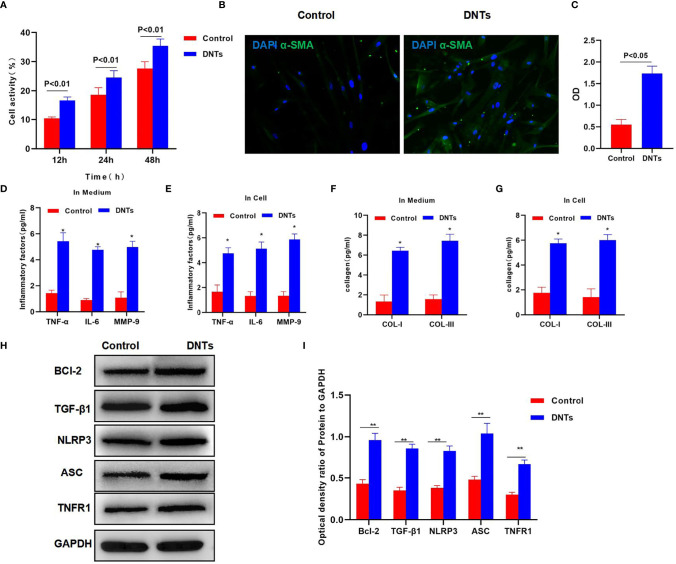
Effect of DNTs on HSC activation and the mechanism. **(A)** DNTs promoted HSC proliferation. The cell viability of DNT group was significantly higher than that of control group. p < 0.01 between groups. **(B, C)** The fluorescence intensity of HSC activation marker α-SMA in DNT group was markedly higher than that in control group. p < 0.01 between groups. **(D, E)** The expression levels of secreted inflammatory factors in cells and the culture medium. The intracellular and secreted inflammatory factor levels in DNT group were markedly higher than those in control group. DNTs promoted the expression of intracellular inflammatory factors. ^*^p < 0.05 compared with control group. **(F, G)** DNTs promoted collagen expression in HSCs. The expression levels of intracellular and secreted COL-I and COL-III markedly increased. ^*^p < 0.05 compared with control group. **(H, I)** DNTs promoted Bcl-2 protein expression, activated TNFR1 and NLRP3, and evidently upregulated protein levels. **p < 0.01 between groups. DNTs, double-negative T cells; HSC, hepatic stellate cell.

Splenic DNTs extracted from liver fibrosis mice were co-cultured with HSCs. The results discovered that DNTs promoted HSC proliferation. Meanwhile, cell viability assay indicated that the cell viability of the DNT co-culture group was significantly upregulated, higher than that of the control group (**Figure 2A**). Meanwhile, α-SMA staining results also revealed that α-SMA expression was upregulated in the DNT group, and fluorescence intensity significantly increased (**Figures 2B, C**). Also, it was discovered in inflammatory factor detection that DNT co-culture increased the expression levels of inflammatory factors in the medium, which were significantly higher than those of the control group. At the same time, the levels of inflammatory factors in cells also increased, suggesting that DNTs promoted the expression of inflammatory factors in HSCs (**Figures 2D, E**). Additionally, collagen detection revealed that DNTs promoted the expression of COL-I and COL-III, and their levels significantly increased in cells and the culture medium, higher than those in the control group (**Figures 2F, G**). As found from protein detection, DNTs promoted TNFR1 expression and NLRP3 activation; meanwhile, the expression of cell anti-apoptotic protein Bcl-2 increased, indicating that DNTs promoted NLRP3 and TNFR1 activation in HSCs (**Figures 2H, I**).

### Effect of Double-Negative T Cells on Hepatic Stellate Cell Activation in HSC-*NLRP3^−/−^*

**Figure 3:**
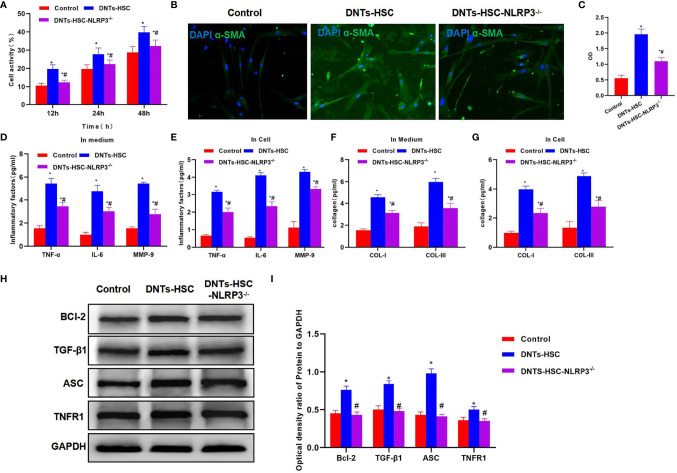
Effect of DNTs on HSC activation after NLRP3 knockdown (HSC-*NLRP3^−/−^*). **(A)** After NLRP3 knockdown, DNTs had weakened ability to enhance HSC viability, which significantly decreased compared with HSC group, but were still higher than those in control group. ^*^p < 0.05 compared with control group, ^#^p < 0.05 compared with DNT-HSC group. **(B, C)** After NLRP3 knockdown, the fluorescence intensity of HSC activation marker α-SMA significantly decreased, and the difference was significant as compared with HSC group. ^*^p < 0.05 compared with control group, ^#^p < 0.05 compared with DNT-HSC group. **(D, E)** As revealed by inflammatory factor expression results, the inflammatory factor levels in HSC-*NLRP3^−/−^* group significantly decreased, lower than those in DNT-HSC group. Similar results were obtained for secreted inflammatory factors. ^*^p < 0.05 compared with control group, ^#^p < 0.05 compared with DNT-HSC group. **(F, G)** After NLRP3 knockdown, DNTs had decreased ability to induce collagen expression in HSCs. The levels of COL-I and COL-III significantly decreased in HSC-*NLRP3^−/−^*, lower than those in DNT-HSC. ^*^p < 0.05 compared with control group, ^#^p < 0.05 compared with DNT-HSC group. **(H, I)** After NLRP3 knockdown, DNTs had reduced ability to induce TNFR1 and Bcl-2 expression, and the protein levels were evidently lower than those of DNT-HSC group. ^*^p < 0.05 compared with control group, ^#^p < 0.05 compared with DNT-HSC group. DNTs, double-negative T cells; HSC, hepatic stellate cell.

After NLRP3 knockdown in HSCs, DNTs were co-cultured with HSC-*NLRP3^−/−^*, which significantly suppressed the increased viability of HSCs, and the difference was significant as compared with the HSC group (**Figure 3A**). α-SMA immunofluorescence (IF) staining results indicated that after NLRP3 knockdown, DNTs had reduced ability to induce its expression, the protein expression significantly decreased, and the fluorescence intensity was lower than that of the HSC group (**Figures 3B, C**). It was discovered when detecting the intracellular and secreted inflammatory factors that DNTs had reduced ability to induce inflammatory factor expression after NLRP3 knockdown, which was markedly lower than that of the HSC group (**Figures 3D, E**). In addition, it was discovered in collagen detection that after NLRP3 knockdown, DNTs had a reduced ability to induce collagen expression in HSCs (**Figures 3F, G**). Protein detection results revealed that NLRP3 knockdown affected TNFR1 expression, decreased Bcl-2 expression, and downregulated the inflammasome component protein ASC (**Figures 3H, I**).

### Effect of Double-Negative T Cells on Hepatic Stellate Cell Activation After TNFR1 Knockdown (HSC-*TNFR1^−/−^*)

**Figure 4:**
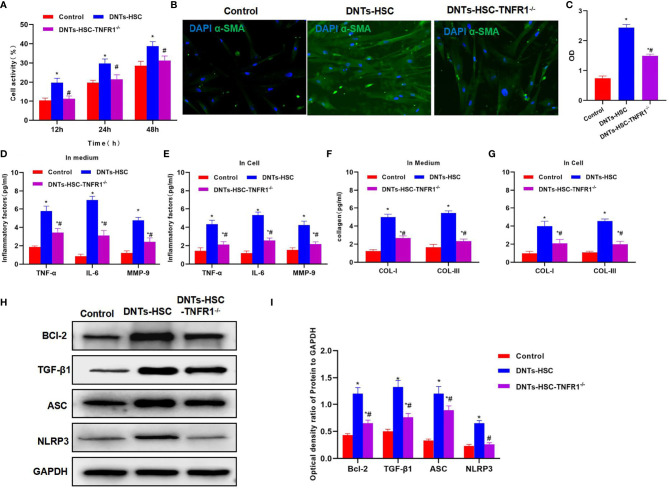
Effect of DNTs on HSC activation after TNFR1 knockdown (HSC-*TNFR1^−/−^*). **(A)** After TNFR1 knockdown, DNTs had weakened ability to induce the upregulation of HSC viability. Cell viability significantly decreased compared with DNT-HSC but was still higher than that of the control group. ^*^p < 0.05 compared with control group, ^#^p < 0.05 compared with DNT-HSC group. **(B, C)** After TNFR1 knockdown, the fluorescence intensity of HSC activation marker α-SMA significantly decreased. ^*^p < 0.05 compared with control group, ^#^p < 0.05 compared with DNT-HSC group. **(D, E)** Results of inflammatory factor expression demonstrated that the inflammatory factor levels significantly decreased in HSC-*TNFR1^−/−^*, lower than those of DNT-HSC group. ^*^p < 0.05 compared with control group, ^#^p < 0.05 compared with DNT-HSC group. **(F, G)** After TNFR1 knockdown, DNTs had reduced ability to induce collagen expression in HSCs. The expression levels of COL-I and COL-III in HSC-*TNFR1^−/−^* markedly decreased, lower than those in DNT-HSC. ^*^p < 0.05 compared with control group, ^#^p < 0.05 compared with DNT-HSC group. **(H, I)** After TNFR1 knockdown, DNTs had reduced ability to induce TNFR1 and Bcl-2 expression, and the protein levels were markedly lower than those of DNT-HSC group. ^*^p < 0.05 compared with control group, ^#^p < 0.05 compared with DNT-HSC group. DNTs, double-negative T cells; HSC, hepatic stellate cell.

TNFR1 is the major receptor of TNF-α, which mediates the activation of downstream NLRP3. After TNFR1 knockdown, DNTs had a weakened ability to induce HSC activation, and the cell viability remarkably decreased as compared with the DNT-HSC group (**Figure 4A**). α-SMA expression significantly decreased in HSC-*TNFR1^−/−^*, and the fluorescence intensity was weakened, suggesting that TNFR1 was related to activation (**Figures 4B, C**). Meanwhile, inflammatory factor and collagen detection revealed that after TNFR1 knockdown, DNTs had reduced ability to induce inflammatory factor expression, secretory inflammatory factors and collagens were also downregulated, and the differences were significant as compared with DNT-HSC (**Figures 4D–G**). TNFR1 also affected NLRP3 activation, and after TNFR1 knockdown, the expression of NLRP3 was suppressed; so was Bcl-2 expression (**Figures 4H, I**).

### Effect of TNF-α Silencing (TNF-α siRNA) in Double-Negative T Cells on Hepatic Stellate Cell Activation

**Figure 5:**
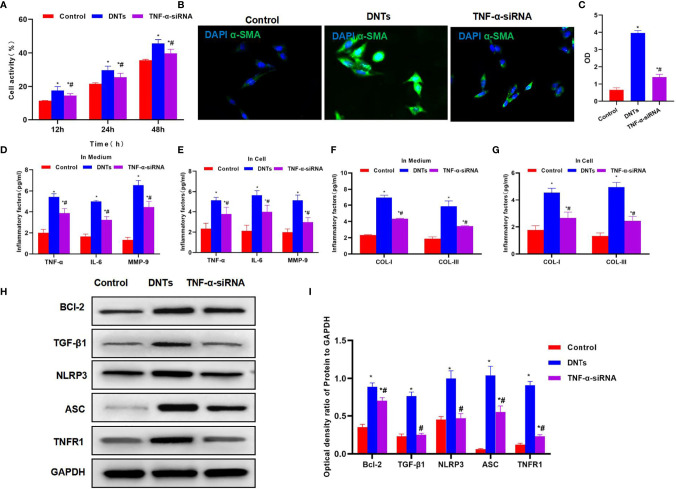
Effect of DNTs on HSC activation after TNF-α silencing (TNF-α siRNA). **(A)** After TNF-α silencing, DNTs had reduced ability to increase HSC viability, and cell viability markedly decreased compared with DNT group. ^*^p < 0.05 compared with control group, ^#^p < 0.05 compared with DNT-HSC group. **(B, C)** Fluorescence intensity of HSC activation marker α-SMA significantly decreased in TNF-α siRNA. ^*^p < 0.05 compared with control group, ^#^p < 0.05 compared with DNT-HSC group. **(D, E)** Expression results of inflammatory factors suggested that the inflammatory factor levels in TNF-α siRNA significantly decreased, lower than those in DNT group. ^*^p < 0.05 compared with control group, ^#^p < 0.05 compared with DNT-HSC group. **(F, G)** After TNF-α silencing, DNTs had reduced ability to induce collagen expression in HSCs. The levels of COL-I and COL-III significantly decreased in TNF-α siRNA, which were lower than those of DNT group. ^*^p < 0.05 compared with control group, ^#^p < 0.05 compared with DNT-HSC group. **(H, I)** After TNF-α silencing, DNTs had decreased ability to induce TNFR1 and Bcl-2 expression, and protein levels were markedly lower than those in DNT group. ^*^p < 0.05 compared with control group, ^#^p < 0.05 compared with DNT-HSC group. DNTs, double-negative T cells; HSC, hepatic stellate cell.

After siRNA was used to silence TNF-α in DNTs, the TNF-α siRNA DNTs were co-cultured with HSCs. The results suggested that DNTs had a weakened ability to promote HSC viability, and the difference was significant as compared with DNTs (**Figure 5A**). TNF-α siRNA DNTs had a weakened influence on α-SMA expression, and the fluorescence intensity remarkably decreased, lower than that of DNTs (**Figures 5B, C**). After TNF-α silencing, the expression and secretion of inflammatory factors in HSC were weakened, and collagen expression also decreased (**Figures 5D–G**). After TNF-α silencing, TNF-α siRNA DNTs had a weakened effect on NLRP3 activation and TNFR1 expression, which were significantly lower than those in the DNT group, and Bcl-2 expression also decreased (**Figures 5H, I**).

### Effect of Double-Negative T Cells on Mouse Liver Fibrosis

**Figure 6:**
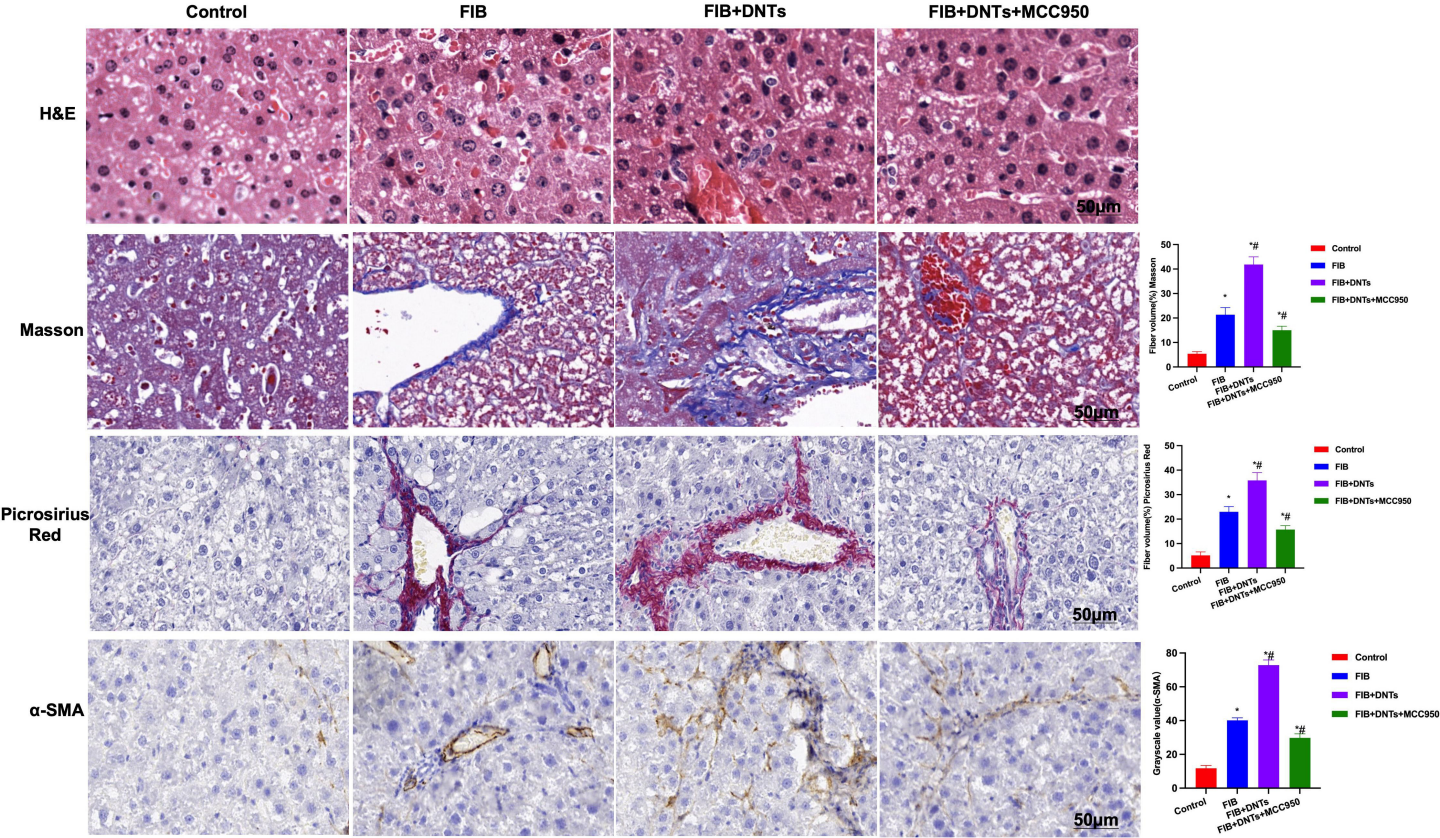
H&E staining indicated obvious fibrosis lesion in FIB mice and apparent tissue inflammation, which were further aggravated in DNTs, along with severe liver injury, while MCC950 improved liver fibrosis degree. Masson and sirius-red staining results indicated that, DNTs promoted liver fibrosis in mice, the expression of collagen fibers and collagens further increased in hepatic tissues, higher than that in FIB, while MCC950 suppressed such changes. Moreover, IHC also revealed that DNTs promoted a-SMA expression. *P<0.05 compared with Control group, #P<0.05 compared with FIB group.

**Figure 7:**
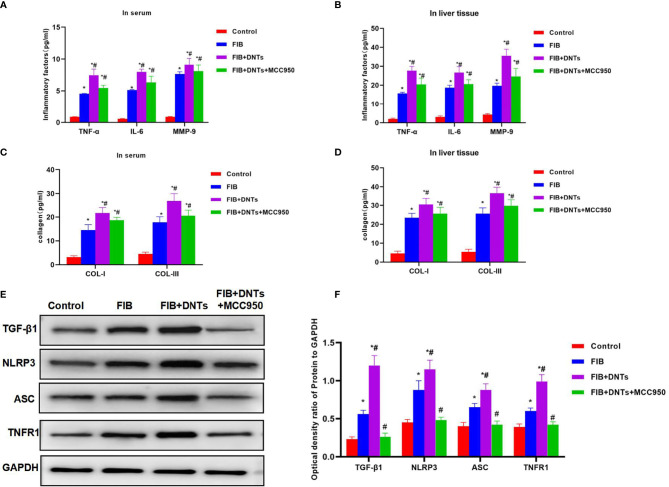
Effect of DNTs on collagens, inflammatory factors, and NLRP3 inflammasome in mice with liver fibrosis. **(A, B)** The expression of inflammatory factors in peripheral blood and hepatic tissues of FIB group significantly increased; DNTs further promoted the expression of inflammatory factors, which was significantly higher than that of FIB group. MCC950 decreased the expression of inflammatory factors. ^*^p < 0.05 compared with control group, ^#^p < 0.05 compared with FIB group. **(C, D)** Expression of COL-1 and COL-III in peripheral blood and hepatic tissues of mice with liver fibrosis was significantly higher than that in control group, while DNTs further promoted collagen expression. ^*^p < 0.05 compared with control group, ^#^p < 0.05 compared with FIB group. **(E, F)** Expression of NLRP3 and TNFR1 increased in FIB, DNTs further promoted their expression, and MCC950 reduced their expression. ^*^p < 0.05 compared with control group, ^#^p < 0.05 compared with FIB group. DNTs, double-negative T cells.

As discovered in pathological changes in mice with liver fibrosis (FIB), FIB mice experienced obvious fibrosis, inflammation, and fibrosis lesion in hepatic tissues. Masson staining suggested obvious collagen fiber expression, which was significantly different compared with the control group. Meanwhile, sirius-red staining also revealed the upregulation of collagens, and α-SMA expression also increased in FIB, significantly higher than that of the control group. DNT injection further aggravated liver fibrosis in mice. Both Masson and sirius-red staining indicated significant expression of collagen fibers and collagens and further upregulation of α-SMA, with a significant difference, as compared with FIB. When NLRP3 inhibitor MCC950 was added at the same time, the fibrosis degree in mice was mitigated, and the difference was significant as compared with DNT intervention (**Figure 6**). As further found from inflammatory factor and collagen detection in mouse serum and hepatic tissues, DNTs further upregulated the expression of inflammatory factors and collagens, and consistent expression was observed between serum and hepatic tissues (**Figures 7A–D**). Protein detection also revealed that DNTs promoted NLRP3 activation and increased TNFR1 expression (**Figures 7E, F**).

## Discussion

DNTs are cells with unique biological functions, and the proportions of DNTs in peripheral blood of healthy subjects and mice are low (about 1%–2%) (16). As discovered in functional research, DNTs suppressed the functions of CD8^+^ T and CD4^+^ T cells (17); meanwhile, TCR^+^ double-negative NKT cells can induce immune tolerance (18, 19). It is found in research on nerve diseases that DNTs can promote microglial cell activation in mice with ischemic stroke to exert the pro-inflammatory effect, and such effect was related to TNF-α secretion (4, 5). DNTs are also the immune cells that express high levels of TNF-α. However, they are rarely investigated, and their functions remain to be further revealed. An imbalance of immune cells to a certain degree is detected in liver fibrosis. Some research has recently discovered Treg/Th17 imbalance (20). Our study suggested that the proportion of DNTs in liver fibrosis significantly increased, higher than that in normal subjects by several folds. DNTs in normal subjects also express high levels of TNF-α, but the expression levels of DNTs TNF-α in liver fibrosis are even higher, indicating that DNTs in liver fibrosis may exert their effect *via* TNF-α (21).

HSCs are the major source of ECM, which plays a critical role in liver fibrosis (22). When various pathogenic factors induce liver injury, HSCs will be activated and differentiated into myofibroblastic-like cells (MFLCs). MFLCs have contractility, pro-inflammatory activity, and pro-fibrosis effect (23) and can secrete multiple ECM components like α-SMA and collagen (24). Research indicates that the activation of NLRP3 inflammasome in HSCs can increase the number of α-SMA-positive cells (25), while positive α-SMA expression is the key marker of HSC activation, which is closely related to liver fibrosis progression (26). In addition, suppressing NLRP3 inflammasome alleviates liver inflammatory response and reduces the expression of collagens and tissue inhibitor of metalloproteinase-1 (TIMP1) (27). Therefore, the activation of NLRP3 inflammasome can activate HSCs, thus resulting in the occurrence and development of liver fibrosis. The activation of NLRP3 is also regulated by TNF-α (28). TNFR1 is the major membrane receptor of TNF-α, and its activation further promotes the activation of downstream NF-κB and inflammasome (29) and increases inflammatory factor expression, while the TNF-α-mediated inflammatory response is also one of the important causes of liver fibrosis progression. As we discovered high expression of TNF-α in DNTs based on human samples, we co-cultured DNTs with HSCs and discovered that DNTs did not obviously damage HSCs; instead, they promoted the cell viability of HSCs, enhanced HSC activation, significantly increased α-SMA expression, and increased the expression of intracellular inflammatory factors as well as collagens COL-I and COL-III. This proved that DNTs promoted HSC activation. Further investigation discovered that DNTs activated TNFR1 and NLRP3 expression in HSCs. According to previous reports, NLRP3 is an important signal that promotes DNT activation, while DNTs probably secrete TNF-α to mediate NLRP3 activation. We knocked down TNFR1 and NLRP3 expression in HSCs; as a result, TNFR1 and NLRP3 knockdown affected the effect of DNTs, suggesting that DNTs indeed activated TNFR1-NLRP3. To verify that DNTs exerted their effect *via* TNF-α, we silenced TNF-α expression in DNTs with siRNA. The results suggested that HSC activation was suppressed; meanwhile, TNFR1 and NLRP3 expression was also inhibited. Therefore, we verified the mechanism that DNTs secreted TNF-α to promote TNFR1-NLRP3 and HSC activation. In animal experiments, we also confirmed that DNTs promoted liver fibrosis progression and significantly increased collagen and collagen fiber expression in cells, while MCC950 (NLRP3 inhibitor) treatment suppressed the effect of DNTs.

## Conclusion

This study discovered that the proportion of DNTs in peripheral blood of liver fibrosis patients significantly increased and that DNTs secreted TNF-α to promote the activation of the TNFR1-NLRP3 axis and regulate HSC activation, thus leading to liver fibrosis progression.

## Data Availability Statement

The original contributions presented in the study are included in the article/supplementary material. further inquiries can be directed to the corresponding author.

## Ethics Statement

The studies involving human participants were reviewed and approved by Jiaxing University. Written informed consent to participate in this study was provided by the participants’ legal guardian/next of kin. The animal study was reviewed and approved by Jiaxing University.

## Author Contributions

Design and operation of the experiment, operation of animal experiment, and data processing: YY, YS, and JW. Collection of clinical samples and detection of inflammatory factors: XZ, WL, and CZ. The proposal of the subject, the design of the experimental process, and the whole process guidance: LG and CH. All authors listed have made a substantial, direct, and intellectual contribution to the work and approved it for publication.

## Funding

This study was funded by Zhejiang Provincial Natural Science Foundation [LYY20H280005].

## Conflict of Interest

The authors declare that the research was conducted in the absence of any commercial or financial relationships that could be construed as a potential conflict of interest.

## Publisher’s Note

All claims expressed in this article are solely those of the authors and do not necessarily represent those of their affiliated organizations, or those of the publisher, the editors and the reviewers. Any product that may be evaluated in this article, or claim that may be made by its manufacturer, is not guaranteed or endorsed by the publisher.
