# Corrigendum: Double-negative T cells regulate hepatic stellate cell activation to promote liver fibrosis progression *via* NLRP3

**DOI:** 10.3389/fimmu.2023.1340576

**Published:** 2023-12-01

**Authors:** Yi Yang, Yongjia Sheng, Jin Wang, Xiaohong Zhou, Wenyan Li, Caiqun Zhang, Li Guo, Chenyang Han

**Affiliations:** ^1^ Department of Pharmacy, The Second Affiliated Hospital of Jiaxing University, Jiaxing, China; ^2^ Department of Neurology, The Second Affiliated Hospital of Jiaxing University, Jiaxing, China; ^3^ Department of Center Laboratory, The Second Affiliated Hospital of Jiaxing University, Jiaxing, China

**Keywords:** double-negative T cells, liver fibrosis, NLRP3, TNFR1, hepatic stellate cells

In the published article, there was an error in **Figure 6** as published. In our four staining methods, the magnification is inconsistent, and the scale annotation is not consistent with the original image. Therefore, we uniformly choose 50μm Display at magnification. The corrected **Figure 6** and its caption appear below.

**Figure 6:**
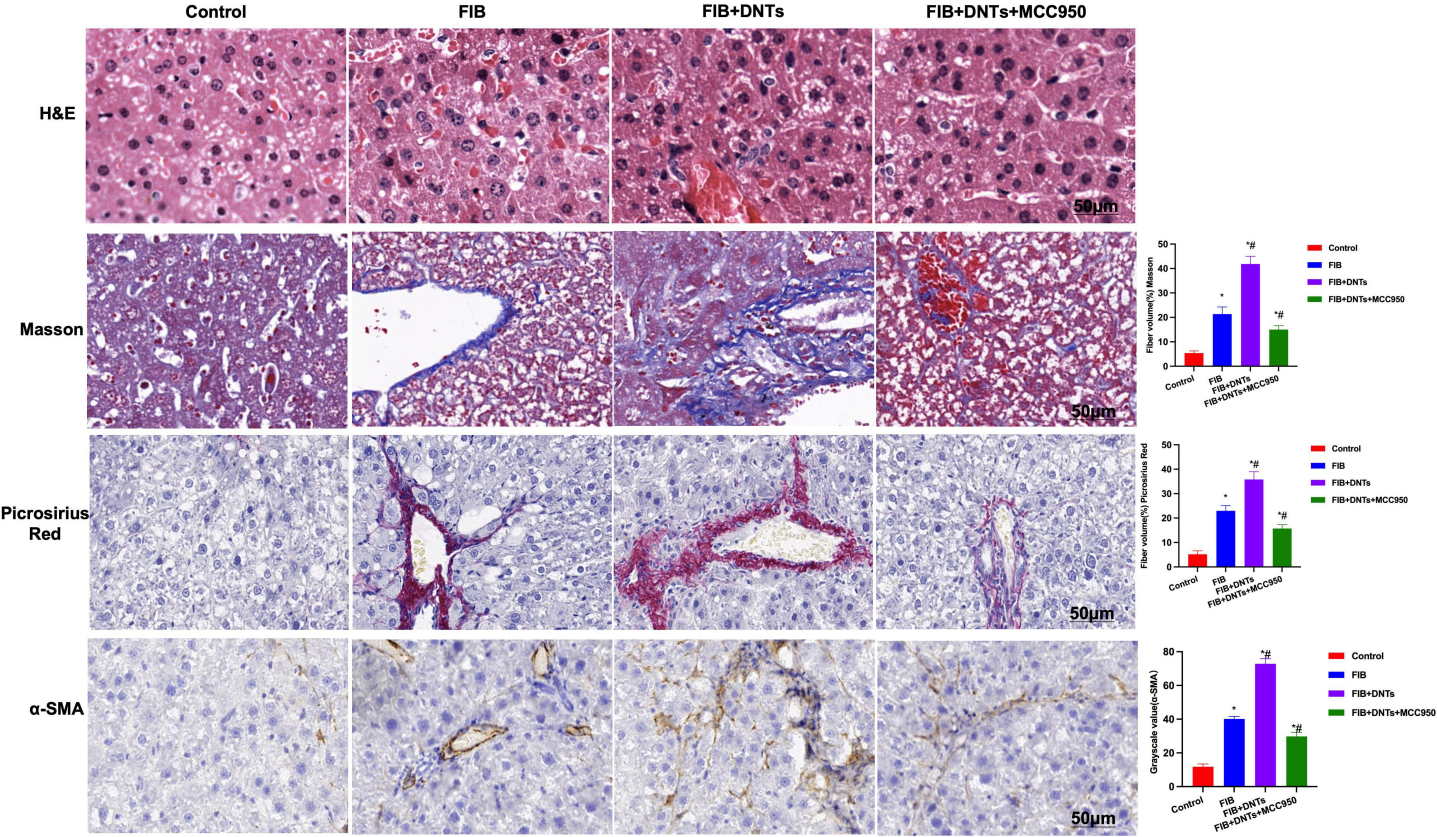
H&E staining indicated obvious fibrosis lesion in FIB mice and apparent tissue inflammation, which were further aggravated in DNTs, along with severe liver injury, while MCC950 improved liver fibrosis degree. Masson and sirius-red staining results indicated that, DNTs promoted liver fibrosis in mice, the expression of collagen fibers and collagens further increased in hepatic tissues, higher than that in FIB, while MCC950 suppressed such changes. Moreover, IHC also revealed that DNTs promoted α-SMA expression. ^*^P<0.05 compared with Control group, ^#^P<0.05 compared with FIB group.

The authors apologize for this error and state that this does not change the scientific conclusions of the article in any way. The original article has been updated.

